# A long-term anti-inflammation markedly alleviated high-fat diet-induced obesity by repeated administrations of overexpressing IL10 human umbilical cord-derived mesenchymal stromal cells

**DOI:** 10.1186/s13287-022-02935-8

**Published:** 2022-06-17

**Authors:** Liudi Wang, Tianyun Gao, Yu Li, Yuanyuan Xie, Sheng Zeng, Chenxu Tai, Yirui Feng, Pingping Shen, Bin Wang

**Affiliations:** 1 Clinical Stem Cell Center, The Affiliated Drum Tower Hospital of Nanjing University Medical School, Nanjing, 210000 China; 2 State Key Laboratory of Pharmaceutical Biotechnology and the Comprehensive Cancer Center, School of Life Science, The Affiliated Drum Tower Hospital of Nanjing University Medical School, Nanjing University, Nanjing, Jiangsu Province China; 3 College of Life Sciences, Nanjing Normal University, Nanjing, 210023 China

**Keywords:** Obesity, MAPK, Adipose tissue, Anti-inflammation, mesenchymal stromal cells (MSCs), IL10, Gene modification, Stem cell therapy

## Abstract

**Objectives:**

Obesity is a chronic process and could activate various inflammatory responses, which in turn aggravates obesity and related metabolic syndrome. Here we explored whether long-term inhibition of inflammation could successfully alleviate high-fat diet (HFD)-induced obesity.

**Methods:**

We constructed stable overexpressing interleukin 10 (IL10) human umbilical cord-derived mesenchymal stromal cells (HUCMSCs) which repeatedly were applied to obesity mice with HFD feeding to obtain a long-term anti-inflammation based on the prominent anti-inflammation effects of IL10 and immunomodulatery effects of HUCMSCs. Then we monitored the features of obesity including body weight, serum ALT, AST, and lipids. In addition, glucose homeostasis was determined by glucose tolerance and insulin sensitivity tests. The infiltrated macrophages in adipose tissues and hepatic lipid accumulation were detected, and the expressions of adipogenesis and inflammatory genes in adipose tissues were examined by real-time (RT) PCR and western blot analysis.

**Results:**

Compared with HUCMSCs, IL10-HUCMSCs treatment had much better anti-obesity effects including body weight reduction, less hepatic lipids accumulation, lower amount and size of adipocyte, greater glucose tolerance, less systemic insulin resistance, and less adipose tissue inflammation in HFD feeding mice. Finally, IL10-HUCMSCs could decrease the activation of MAPK JNK of adipose tissue induced by HFD. The inhibition of MAPK JNK signal pathway by a small chemical molecule SP600125 in 3T3-L1 cells, a preadipocyte line, reduced the differentiation of adipocytes and lipid droplet accumulation.

**Conclusion:**

A lasting anti-inflammation based on gene modified stem cell therapy is an effective strategy in preventing diet-induced obesity and obesity-related metabolic syndrome.

## Introduction

Obesity is a global health problem, which is mainly caused by the imbalance between energy intake and consumption [1]. Accumulating researches have showed that obesity was associated with chronic systemic low-grade inflammation and also considered to be one of the major risk factors for chronic disorders such as kidney disease, nonalcoholic fatty liver disease, type 2 diabetes, and cardiovascular diseases [2–5]. Obesity and its related chronic inflammation are closely associated with various metabolic syndromes, but the underlying mechanism is still elusive. Adipose tissue as the first site that has been proved to be related to inflammation and obesity mediates the inflammatory response by secreting adipokines, inflammatory factors, and chemokines [6]. These secretory factors(such as IL6, IL1β, TNFα, MCP-1, etc.) can enhance the recruitment of immune cells (especially macrophages) to adipose tissue, thereby in turn aggravating adipose tissue and systemic inflammation [7, 8]. For example, IL6, a pro-inflammatory factor, is associated with the obesity-induced insulin resistance [9]. In turn, low-grade chronic adipose tissue inflammation is associated with excess body fat mass and is characterized by the infiltration of macrophages in adipose tissue which would polarize into pro-inflammatory M1 state, so as to synthesize more pro-inflammatory factors and chemokines, leading to more severe obesity [10, 11].Adipose tissue macrophages (ATMS) in inflammation play an important role in altering the characteristics of obesity [6]. In both humans and animals, ATMs accumulated in adipose tissue caused excessive hypertrophy of adipose tissue, an increased weight gain, hepatic lipids accumulation, glucose intolerance, and insulin resistance [12, 13]. Specifically, low-grade chronic inflammation in adipose tissue is associated with insulin resistance, which promotes the development of type 2 diabetes in obesity individuals [14]. The association between obesity, adipose tissue inflammation, and metabolic disease makes anti-inflammatory strategies an effective therapy in ameliorating obesity. Therefore, it is speculated that blocking the obesity-related systemic low-grade inflammation could alleviate diet-induced obesity metabolic syndrome [15, 16].

Interleukin10 (IL10) is a representative anti-inflammatory cytokine secreted by lymphocytes, including T cells, B cells, natural killer cells, dendritic cells, and mast cells. It can not only reduce the expression of immune receptor MHC-II, but also inhibit the production and release of pro-inflammatory factors such as IL2, IFN-γ, IL6, and tumor necrosis factor-α (TNFα) [17]. Therefore, IL10 plays an important role in controlling inflammation and modulating immune responses, which can well contradict the inflammation in adipose tissue and insulin resistance in obesity [16, 18, 19]. Gotoh K et al. reported [20–22] that HFD-induced obesity decreased the serum levels of IL10 in animals, and the IL10 reduction enhanced the inflammation in several organs. The spleen-derived IL10 could not only downregulate the severity of HFD-induced nonalcoholic fatty pancreas disease, but also might protect against the development of chronic kidney disease. Furthermore, IL10 shares a similar structure and function with leptin, and over-expression of murine IL10 in skeletal muscle by adeno-associated virus (AAV) transfer meliorated hyperphagia, obesity, glucose intolerance, and insulin resistance in leptin deficiency mice by substituting for leptin [23]. Consistent with animal model, patients with impaired glucose tolerance and type 2 diabetes also had a lower plasma concentration of IL10 [24].Therefore, it is speculated that the administration of exogenous or endogenous IL10 could be an effective strategy for treating obesity and metabolic syndromes induced by HFD.

Mesenchymal stromal cells (MSCs) are a kind of adult stem cells derived from mesoderm. They were first found in bone marrow and could also be isolated from a variety of tissues such as umbilical cord, fat, umbilical cord blood, amniotic fluid, and tooth [25, 26]. MSCs not only have the ability of self-replication and multidirectional differentiation potentials, but also have obvious immune regulation functions. To date, it has been reported that under the stimulation of different inflammatory cytokines, MSCs could migrate to damaged tissues and release a variety of factors including EGF, BFGF, HGF, Ang-1, etc., to enhance angiogenesis, inhibit leukocyte migration, and initiate stem cell differentiation to promote tissue regeneration and repair [27, 28]. In recent years, studies have shown that MSCs infusion produced significant antidiabetic effects and ameliorated insulin sensitivity in T2D rats [29], and reduced the exogenous insulin requirement, increased glucose tolerance, and improved the insulin sensitivity index in T2D patients [30]. Moreover, the therapeutic potentials of MSCs could be strengthened by integrating functional genes or preconditioning for various diseases [10, 12, 13]. For example, in our recent study, we introduced BFGF into HUCMSCs, which could significantly enhance the neuronal regeneration and locomotion function recovery in mice with completely transected spinal cord injury [31]. There is few research to investigate whether MSCs genetically modified with anti-inflammation genes could alleviate the obesity and metabolic disorders induced by HFD.

IL10 protein has a short half life, lasting for only a few hours in vivo [32], and thus it needs lasting administration when applied to treat corresponding diseases. Based on the chemotaxis of MSCs to injured sites, MSCs could be a good carrier candidate for gene therapy. In this study, we established overexpressing IL10 HUCMSCs (IL10-MSCs) and intravenously injected into mice with HFD feeding to investigate their effects on obesity and metabolic disorders. Results showed that IL10-MSCs could significantly counteract the HFD-induced obesity-related metabolic symptoms including body weight gain, hepatic and adipocyte lipids accumulation, glucose tolerance, insulin resistance, and serum lipid indicators, compared with unmodified MSCs. As expected, IL10-MSCs administration also alleviated the low-graded activation of MAPK JNK signal induced by HFD, which regulated adipocyte differentiation and lipid droplet accumulation. Thus, our findings revealed that IL10- MSCs provided an effective method to resist the inflammatory responses and metabolic disorder caused by HFD feeding, indicating that inhibition of obesity-related inflammation based on cell therapy was an effective strategy in preventing obesity and obesity-related metabolic syndrome.

## Materials and methods

In this study, all procedures involving human subjects were approved by the Research Ethics Board of Nanjing Drum Tower Hospital (Approval No. GCP-SCP/17/2).

### Establishment of IL10-MSCs

Clinical-grade HUCMSCs were used in this study which was greatly optimized in our previous research [33].The entire process including isolation, cultivation, identification, quality control, and storage was conformed to GMP quality standards. After evaluation, qualified clinical HUCMSCs derived from three umbilical cords of female newborns in second generation were mixed and resuscitated for following experiments to avoid the individual heterogeneity [34].

**Fig. 1:**
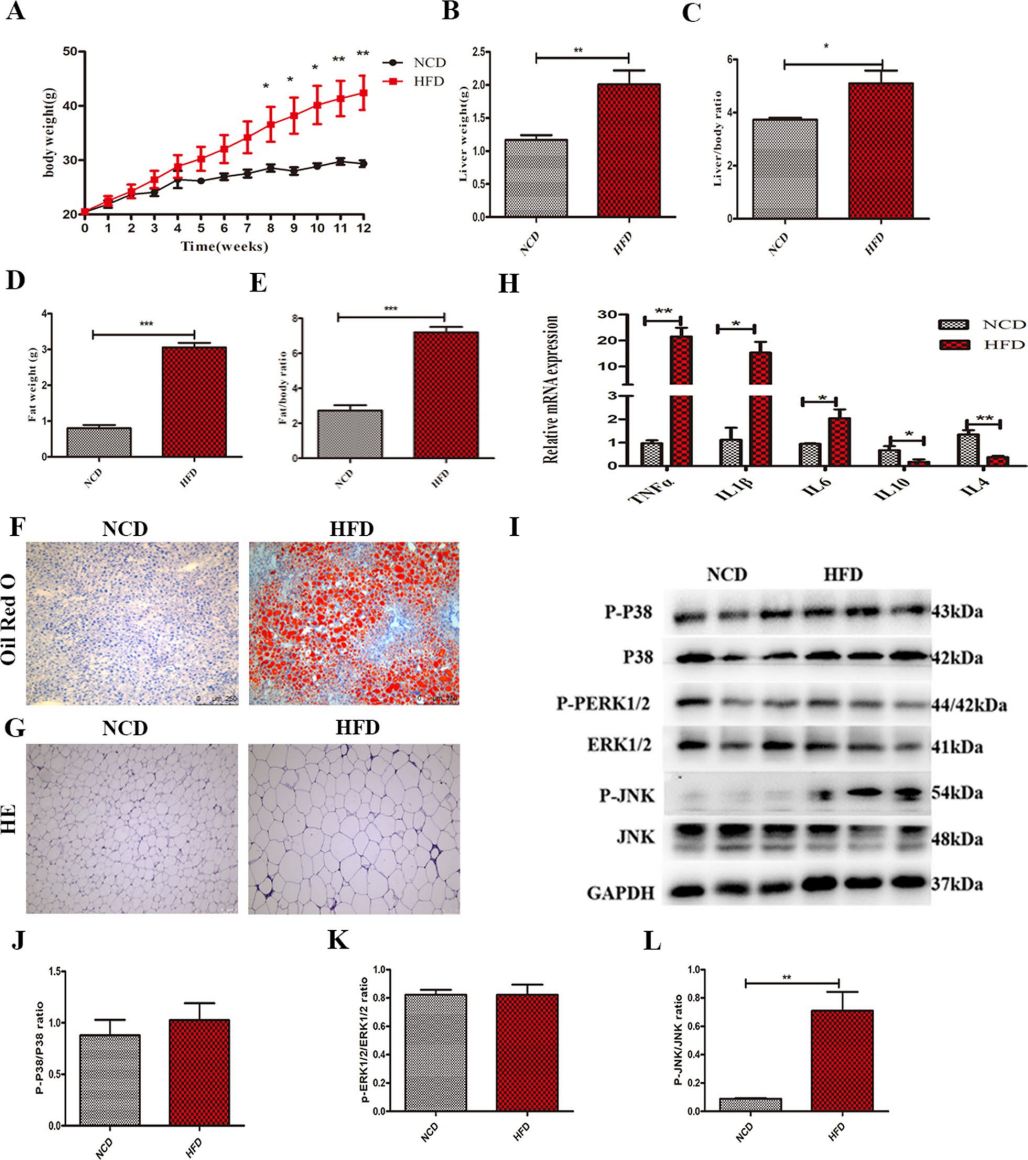
HFD-induced obesity caused systemic inflammation. **A** Mice were fed with NCD (11.6% kcal fat) or HFD (60% kcal fat) for 12 weeks, and body weights were measured weekly. HFD significantly induced body weight gain compared with NCD feeding. **B-C** The weights of liver tissues and liver/body weight ratio were obviously increased by HFD feeding. **D-E** The fat gains and fat/body weight ratio of HFD group were significantly increased compared with NCD group. **F** Oil Red O staining of liver sections was carried out. HFD feeding led to obvious lipid droplet accumulation. **G** The H&E of epididymal white adipose tissues. **H** The expressions of inflammatory cytokines were revealed by RT-PCR. **I** The phosphorylations of MAPK p38, ERK1/2, and JNK in visceral white adipose tissues were detected by western blot analysis. **J-L** The quantified data of MAPK p38, ERK1/2 and JNK phosphorylation were analyzed by Image J, respectively. (N = 6 per group). (* *p* < 0.05; ** *p* < 0.01)

The human IL10 gene (Ref sequence: NM_000572.2) was integrated into vector pCMV/hygro for high-level stable expression in mammalian hosts. The IL10 recombinant vector was purified with QIAGEN Endofree plasmid kit (Cat.12362, QIAGEN, Hilden, Germany) and linearized with the restriction enzyme NruI (Cat.1168A, Takara Biomedical Technology (Beijing) Co., Ltd.) for electro-transformation. The clinical-grade HUCMSCs at passage 2 (P2) was performed electro-transformation. The transfected cells were divided into blank control plasmid (NC) and recombinant IL10 plasmid groups. After electro-transformation, 100 μg/ml hygromycin was used for stable clone screening. After 14 days, cell colonies were picked up under stereomicroscope (K-400L, MOTIC CHINA GROUP CO., LTD.) for expansion culture and each colony represents a cell line. When each clone culture grew to 90% confluence, the supernatant was harvested and IL10 secretion level was detected by ELISA KIT (70-EK110/2–96, MultiSciences). Then, the stably expressing IL10 clones were expanded into P5-P6 generation and were frozen as seeding cells. The negative control HUCMSCs were also obtained and cryopreserved in the same generation. Among multiple clones, we found that one of screened overexpressing IL10-MSCs cones had a good growth potential and secreted a high level of IL10 (Fig. 1B). Then the IL10-MSCs were resuscitated and passaged about 1–2 generations for subsequent experiments in vivo and in vitro.

### Surface marker expressions of IL10-MSCs by flow cytometric analysis

Flow cytometric analysis was performed to identify the surface marker expressions of IL10-MSCs. The cells were collected by enzymatic digestion, and 1 × 10^6^ cells were incubated with CD45-FITC, CD34-FITC, CD90-FITC, CD44-PE, CD19-PE, CD105-PE, CD73-PE, and HLA-DR-PE antibodies (BD, San Diego, CA, USA) for 15 min, respectively. After washing with PBS three times, a FACScan (BD FACS Aria™; NJ, USA) and Flow Jo V10 software were used to identify and analyze the surface marker expressions of the cells.

### IL10-MSCs labeling and homing experiments in vivo

IL10-MSCs were labeled by dyechloromethylbenzamido-1,1’-dioctadecyl-3,3,3’,3’-tetramethyl indocarbocyanine perchlorate (CM-Dil, Sigma-Aldrich, USA) in accordance with the manufacturer’s guidance. Cells were digested and labeled with CM-Dil solution with concentration of 20 μg/mL. Then, IL10-MSCs mixed suspension was incubated at 37 °C for 15 min and 4 °C for 15 min. To evaluate the distribution of IL10-MSCs in mice at day 3, 6, and14 post-transplantation, the CM-Dil-labeled IL10-MSCs were injected through the tail vein of mice. After cell transplantation, livers and lungs of mice were collected at 3, 6, and 14 days.

### HFD-induced obesity mice model and MSCs treatment

All animal procedures were in accordance with the protocol approved by the animal experimentation committee of the Affiliated Drum Tower Hospital of Nanjing University Medical School. Male 6-week-old C57BL/6 J mice were raised in the center of the experiment animal (temperature at 23 ± 2 °C and air humidity at 40–60%). The room kept dark for 12 h and illuminating for 12 h. Mice (20 ± 1 g) were divided into four groups according to different treatments: normal chow diet (NCD), high-fat diet (HFD), HFD plus MSCs-injected (MSCs) and HFD plus IL10-MSCs-injected (IL10-MSCs) groups. The mice were fed with chow diet: fat, 4.6%; 11.6% kcal from fat; or HFD (fat, 32%; 56.7% kcal from fat, D12492, Research Diets) for 14 weeks. Mice were free to eating and drinking. Body weights of the animals were recorded weekly. MSCs or IL10-MSCs (1 × 10^6^ cells/each mouse) were intravenously injected into a HFD mouse (n = 12 for each group) every 2 weeks.

### Biochemical analyses

The levels of serum biochemical indicators in mice at week 14 after HFD feeding were measured by automatic biochemical analyzer, including triglyceride (TG), total cholesterol (TC), high- and low-density lipoprotein cholesterol (HDL-C and LDL-C), alanine aminotransferase (ALT), and aspartate aminotransferase (AST).

### Liver triglyceride determination

Mouse liver tissues were harvested at week 14 after HFD feeding and homogenized with tissue homogenizer. Then, the supernatant was tested and the concentration of triglyceride was determined according to the manufacturer's instructions of triglyceride colorimetric determination kit purchased from Solarbio (bc0620, China).

### Metabolic cage analysis

At the week 12 after HFD feeding, the energy metabolism of mice was comprehensively monitored by metabolic chamber. Each mouse was caged in one metabolic chamber, and metabolic data were begun to collect after 48 h of adaptation. Oxygen consumption (VO_2_), carbon dioxide production (VCO_2_), and respiratory exchange ratio (RER) were assessed in 36-h single-housed mouse in the PhenoMaster system (TSE Systems, Bad Homburg, Germany).

### Glucose tolerance test (GTT) and Insulin tolerance test (ITT)

GTT was performed on mice at the week 13 after intervention. After fasting for 6 h, the level of basal blood glucose was measured, and then mice were injected intraperitoneally with 2 g glucose/kg body weight (Invitrogen, the Netherlands). These blood samples collected from mouse tail vein were measured for blood glucose level at 15, 30, 60, 90, and 120 min after injection.

At week 14 after HFD feeding, the mice were subjected to ITT. After fasting for 6 h, mice were injected intraperitoneally with 0.75 U insulin solution/kg body weight. The blood samples collected from tail vein were measured for blood glucose level at 0, 15, 30, 60, 90 and 120 min after injection.

### White adipose tissue and liver histology/immunohistochemistry

The mice were killed at week 14 after HFD feeding. The subcutaneous and epididymal white adipose tissues and liver tissues were fixed with 4% paraformaldehyde and embedded in paraffin. Paraffin sections of mice tissues (5 μm) were stained with hematoxylin and eosin (H & E). The size of 300 adipocytes from six mice in each group was measured using Image J software. Some liver tissues were embedded with OCT reagent, sectioned, and stained with Oil Red O for assaying the lipid accumulation. For immunohistochemistry, the sections of paraffin-embedded adipose (5 μm) were deparaffinized, followed by ethanol and PBS serial rehydration, incubated with 3% hydrogen peroxide for 15 min and washed with water to remove endogenous peroxidase. Antigen retrieval was performed in citrate buffer (pH 6.0) for 15 min using a steamer. The cooled sections were blocked in bovine serum albumin, incubated overnight at 2–8 °C with primary antibodies of F4/80(Abcam, USA), and corresponding secondary antibodies (Typng, China). Finally, the sections went through a series of processes including DAB staining, hematoxylin counterstaining, ammonia back blue, ethanol dehydration, xylene transparency, sealing, and microscope photography (Leica microscope, USA). For immunofluorescence staining, the mice with CM-Dil-labeled IL10-MSCs administration were killed at day 3, 6, and 14, and major organs (liver and lung) were obtained and fixed in 4% paraformaldehyde. The tissues were cut into 10-μm-thickness sections using a Lecia CM1950 (Leica Biosystems, Wetzlar, Germany). The cell apoptosis of the transplanted CM-Dil-labeled IL10-MSCs was evaluated by using a one-step TUNEL cell apoptosis detection kit (KeyGen Biotech, China). Images were quantified using Image J software (version 1.4.3.67).

### Reverse transcription-polymerase chain reaction (RT-PCR)

Total RNAs were extracted from adipose tissues using Trizol reagent (Invitrogen, USA) and reverse-transcribed into cDNA using reverse transcription Kit (R323-01, vazyme, China) according to the manufacturer's instructions. RT-PCR was performed with a ChamQ Universal SYBR PCR Master Mix (Q711-02, vazyme, China) by ABI QuantStudio™ 6 Flex system (Thermo Fisher Scientific, MASS, USA). The specific primers are listed in Table 1. The mRNA expression was relatively quantified according to CT value and calculated by 2^−∆∆Ct^ method.

**Table 1:** Primers used for quantitative real-time PCR analysis

Primer name	Species	Sequence forward (5'to3')	Sequence reverse (5'to3')
IL10	Mouse	GCTGAGGCGCTGTCATCGATTT	GGCCCTGCAGCTCTCAAGTGT
IL6	Mouse	TCAATTCCAGAAACCGCTATGA	CACCAGCATCAGTCCCAAGA
IL1β	Mouse	CGTGCTGTCGGACCCATATGAG	GCCCAAGGCCACAGGTATTT
IL4	Mouse	TCACTGACGGCACAGAGCTA	CTGTGGTGTTCTTCGTTGCTG
TNFα	Mouse	ACGTCGTAGCAAACCACCAA	ACCCTGAGCCATAATCCCCT
PPARγ	Mouse	TCGCTGATGCACTGCCTATG	GAGAGGTCCACAGAGCTGATT
C/EBPα	Mouse	CAAGAACAGCAACGAGTACCG	GTCACTGGTCAACTCCAGCAC
SREBP-1c	Mouse	GAGCGAGCGTTGAACTGTAT	ATGCTGGAGCTGACAGAGAA
Adiponectin	Mouse	TGTTCCTCTTAATCCTGCCCA	CCAACCTGCACAAGTTCCCTT
GAPDH	Mouse	ACAACTTTGGCATTGTGGAAG	GTTGAAGTCGCAGGAGACAAC

### Western blot analysis

Mouse adipose tissue was homogenized with a tissue homogenizer at centrifuge of 12000 g at 4 °C for 10 min using RIPA lysis buffer containing protease inhibitor (Beyotime Biotechnology) and phosphatase inhibitor (MedChemExpress).The supernatant was aspirated, and the protein concentration was measured by the BCA Protein Assay Kit (Vazyme Biotech Co., Ltd.).The protein sample was loaded for polyacrylamide gel electrophoresis and transferred to a nitrocellulose membrane in an electrophoresis transfer buffer containing 10% methanol. The membrane was blocked with 5% BSA (Sigma-Aldrich) for 2 h at room temperature, followed by incubation overnight at 4 °C with anti-p38(1:500; Proteintech™), anti-phosphorylation p38(1:1000;Beyotime), anti-ERK1/2(1:2000; Proteintech), anti-phosphorylation ERK1/2(1:1000; Beyotime), anti-JNK(1:3000; Proteintech™), anti-phosphorylation JNK (1:1000; Beyotime), and anti-GAPDH (1:50,000; Proteintech Wuhan, China) antibodies. After overnight incubation, the blots were incubated with the corresponding secondary antibodies (1:10,000; Bioworld) for 1 h at room temperature. Bands of target proteins were visualized using an enhanced chemiluminescence kit (Vazyme Biotech Co.,Ltd).

### 3T3-L1 preadipocytes culture and differentiation

3T3-L1 preadipocytes differentiation was divided into 4 processes. First, 3T3-L1 preadipocytes were cultured in DMEM (low glucose) containing 10% calf serum and 1% penicillin/streptomycin at 37 °C in a 5% CO_2_ incubator. Second, after reaching confluence, the complete medium was changed with adipocyte differentiation medium I (high-glucose DMEM supplemented with 10% FBS, 1% penicillin/streptomycin, 0.5 Mm IBMX, 1 μM DEX, and 10 μg/ml insulin). Third, after 72 h, the medium was replaced with adipocyte differentiation medium II (high-glucose DMEM containing 10% FBS, 1%penicillin/streptomycin, and 10 μg/ml insulin). Fourth, after 48 h of culture, the cells were cultured with high-glucose DMEM containing 10% FBS and 1% penicillin/streptomycin for another 2 days. The 3T3-L1 adipocyte differentiation was assayed using Oil Red O staining. For investigating the role of MAPK JNK in adipocyte differentiation, the JNK inhibitor SP600125 (2 μg/ml) was added into differentiation medium during second and third processes. For investigating the role of MSCs or IL10-MSCs in adipocyte differentiation, the condition medium from MSCs and IL10-MSCs was supplemented with corresponding adipose differentiation additives as complete medium for second and third differentiation processes.

### Statistical analysis

Data were presented as the mean ± standard derivation (SD). *p* < 0.05 was considered as statistically significant difference. The data were analyzed and graphed by GraphPad Prism (version5.00, San Diego, USA).

## Results

### HFD-induced obesity is along with lasting systemic inflammation

To determine whether HFD-induced obesity could lead to a lasting systemic inflammation, we first assessed the weight gain in mice fed the NCD (11.6% kcal fat) and HFD (60% kcal fat) for 12 weeks. After 8 weeks, HFD-fed mice gained significantly more weight gain than the NCD mice (Fig. 1A). Weight gain in obesity is usually accompanied by pathological changes in multiple organs such as liver and WAT [35]. As expected, HFD-fed mice had significantly enlarged liver (Fig. 1B-C) and WAT (Fig. 1D-E) compared with NCD mice. Moreover, we observed HFD feeding significantly increased lipid accumulation in liver (Fig. 1F) and adipocyte hypertrophy(Fig. 1G)**.** Adipose tissue inflammation is closely related to obesity, especially the chronic inflammation in visceral white adipose tissue [36]. In HFD feeding mice, the expressions of pro-inflammatory genes (TNFα, IL1β, and IL6) in WAT were higher than that in NCD mice (Fig. 1H). In sharp contrast, the expression levels of anti-inflammatory genes (IL10 and IL4) were significantly lower compared with NCD mice (Fig. 1H). Mitogen-activated protein kinases (MAPK) signal pathways including p38, ERK1/2, and JNK can regulate cellular inflammatory response, which also have an extremely important impact on metabolic changes and inflammation associated with HFD [37, 38]. We also found that the expressions of inflammatory factors remarkably increased in adipose tissue of obese mice, so we speculated that HFD feeding could activate MAPK signal pathways and played an import role in adipose metabolic changes. We found that the phosphorylation level of JNK, not Erk1/2, and p38 was increased in visceral white adipose tissue of HFD-induce obese mice compared with NCD mice (Fig. 1I-L). These results clearly proved that HFD feeding-induced obesity is along with a long-term inflammation.

### Establishing stably over-expressing IL10-MSCs

**Fig. 2:**
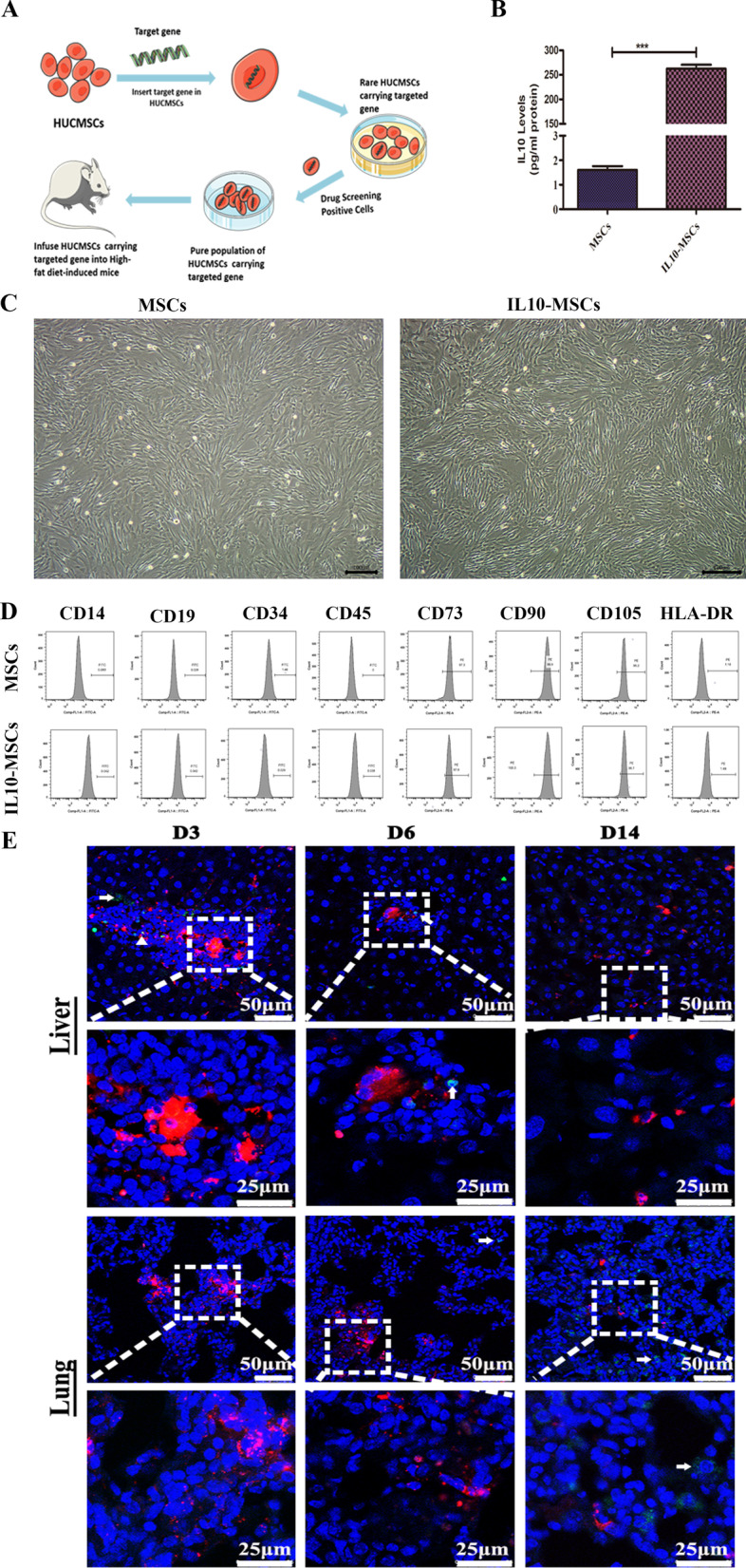
Stable IL10-HUCMSCs were established and the distributions of grafted IL10-MSCs were identified. **A** The process of establishing IL10-MSCs. **B** The analysis of secreted IL-10 by MSCs and IL10- MSCs using ELISA. **C** The morphology of MSCs and IL10-MSCs were observed under a microscope. **D** The surface marker expressions of MSCs and IL10- MSCs were assayed by FCAS analysis. **E** The distribution of CM-Dil-labeled IL10-MSCs (red) in liver and lung at day 3, 6 and14 post-transplantation via intravenous route. (N = 6 per group). Data presented as mean ± SD. ***p < 0.001

The human recombinant IL10 plasmid was transfected into HUCMSCs by using electroporation instruments and nucleofection (Amaxa Kit V, Lonza). After screening using 100 μg/ml hygromycin, positive clones were selected and expanded to stable cell lines (Fig. 2A). Secreted IL10 protein levels by MSCs and IL10-MSCs supernatants were measured by ELISA. The results showed IL10 -MSCs secreted much higher level of IL10 protein than MSCs (Fig. 2B). The quality evaluations of gene-modified MSCs included morphology, surface marker expression, and so on. The morphology of IL10-MSCs did not change compared with MSCs (Fig. 2C). IL10 -MSCs highly expressed surface marker CD105, CD90, and CD73(≥ 95%) and lowly expressed CD14, CD19, CD34, CD45, and HLA-DR (≤ 2%), which was consistent with MSCs (Fig. 2D), indicating IL10-MSCs met the minimal criteria of MSCs suggested in the guidelines from the Mesenchymal and Tissue Stem Cell Committee of the International Society for Cellular Therapy (ISCT) [39]. Thus, MSCs and IL10-MSCs were used for in cell and animal experiments. In order to observe the distribution and survival time of IL10-MSCs in vivo, CM-Dil-labeled IL10–MSCs were infused into mice by intravenous route. As shown in Fig. 2E, grafted IL10-MSCs are accumulated mainly in the liver and lung. The red fluorescence signal intensity annihilated significantly from day 3 to day 14 after infusion. Simultaneously, the cell apoptosis of transplanted CM-Dil-labeled IL10-MSCs at day 3, 6, and 14 was evaluated by using TUNEL staining with green fluorescent probe fluorescein (FITC). The results showed that the red fluorescence of most CM-Dil-labeled IL10-MSCs rarely coincided with the green fluorescence of apoptotic cells. The dotted box represented the red fluorescent of CM-Dil-labeled IL10-MSCs, and the arrows represented the apoptotic host cells, which emitted green fluorescence. The triangles represented the apoptotic transplanted IL10-MSCs, in which the green fluorescence was co-located with the red fluorescence. As shown in Fig. 2E, there were few apoptotic IL10-MSCs. The present findings had proved that some IL10-MSCs could survive in vivo for at least 14 days. Therefore, IL10-MSCs were injected every 2 weeks to treat HFD-induced obesity.

### IL10- MSCs treatment markedly alleviating HFD-induced obesity

**Fig. 3:**
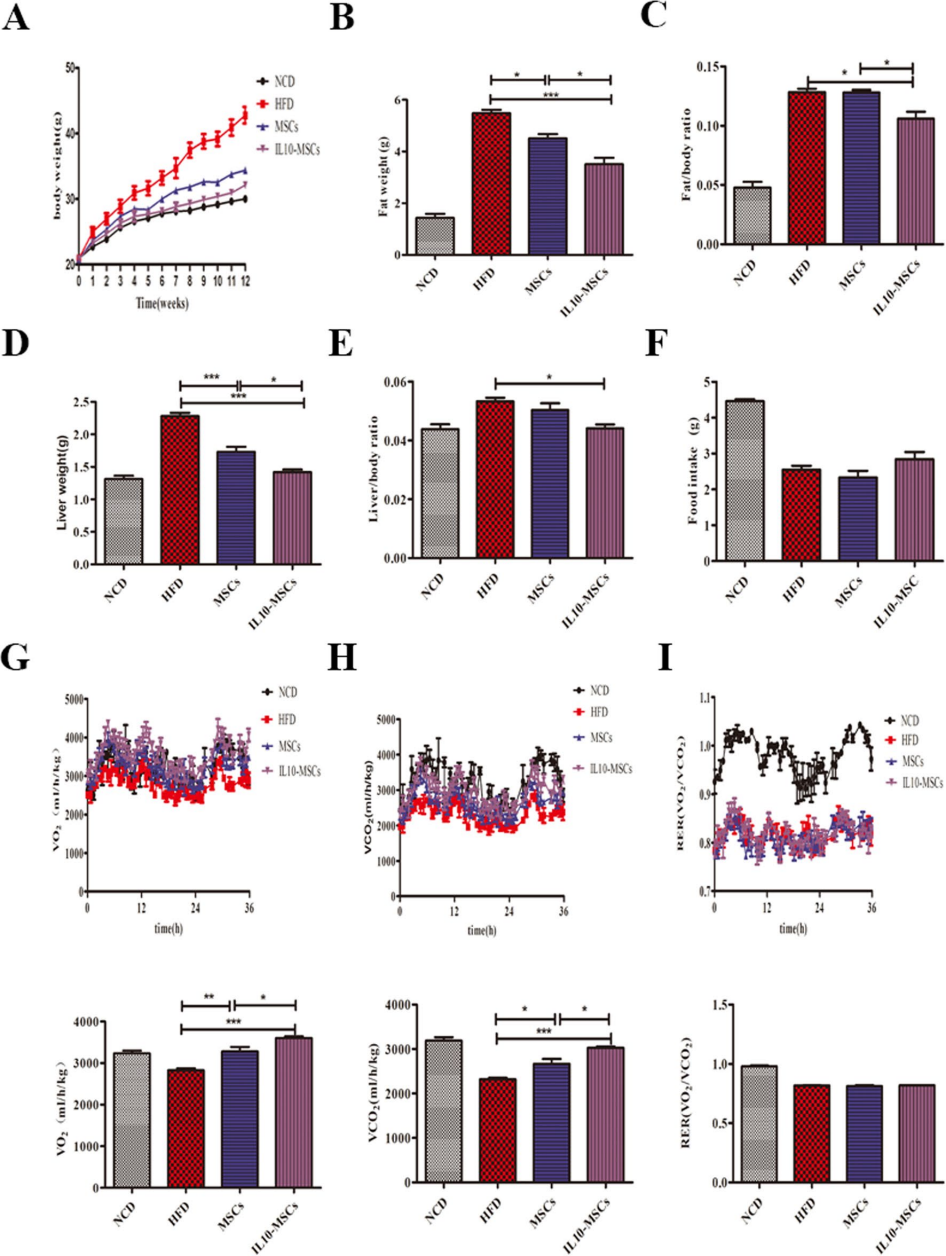
MSCs and IL10-MSCs reduced HFD-induced obesity.**A** Mice were fed with NCD or HFD for 12 weeks, and body weights were measured weekly. Both MSCs and IL10-MSCs treatments could markedly alleviate the HFD-induced obesity, and IL10-MSCs had a better obesity inhibition. **B-C** The weights of subcutaneous and epididymal white adipose tissues were assayed. MSCs and IL10-MSCs significantly inhibited the fat gains and fat/body weight ratio induced by HFD, and the latter had better effect. **D-E** MSCs and IL10-MSCs treatments inhabited the weights of liver tissues and liver/body weight ratio induced by HFD feeding. **F** Food intake was assayed according to metabolic cage analysis, and there were no significant differences among 4 groups. **G-H** The O_2_ consumption and CO_2_ release were evaluated in mice among 4 groups. HFD feeding reduced O_2_ consumptions and CO_2_ release, which were relieved by MSCs and IL10-MSCs treatment. **I** RER value was assayed and there were no significant differences among 4 groups. (N = 6 per group). (* *p* < 0.05; ** *p* < 0.01; ***p < 0.001)

In this study, mice were fed with HFD or NCD and the body weights were recorded every week. The weight growth in HFD group kept a significant increase compared with NCD group while both IL10-MSCs and MSCs treatments could inhibit the HFD-induced weight gain at indicated time points. Especially IL10-MSCs treatment had a better decrease in weight gain compared with MSCs treatment (Fig. 3A). The development of obesity is often accompanied by the increase of fat and liver enlargement [35, 40]. Thus, we also weighed the fat (subcutaneous and epididymal white adipose tissues) and liver tissues to investigate the effects of IL10-MSCs on obesity after the mice were killed at week 14 post-HFD feeding. Both MSCs and IL10-MSCs treatments significantly decreased the weights of fat and liver tissues and, meanwhile, decreased the ratios of fat body and liver body induced by HFD; especially, IL10-MSCs had a better decrease (*p* < 0.05) (Fig. 3B-E). These results indicated that IL10-MSCs had a better effect on alleviating weight gains induced by HFD.

Obesity is mainly caused by the imbalance between energy intake and consumption. Thus we wondered IL10-MSCs treatment whether affected the energy intake or consumption to cover the HFD-induced weight gains. The metabolic cage analysis was carried out, and results showed that there was no significant difference in food intake among 4 groups, indicating that IL10-MSCs did not affect the energy intake to decrease the HFD-induced weight gain (Fig. 3F). Then we further analyzed the mouse respiratory O_2_ consumption and CO_2_ release, and the respiratory exchange rate (RER) (Fig. 3G-I). RER is an important indicator for inferring respiratory substrates, and the respiratory substrates of mice can be judged based on the value of RER. The O_2_ consumption, CO_2_ release, and RER value of mice in 4 groups were recorded when they breathed. Compared with NCD animals, the O_2_ consumption and CO_2_ release of HFD animals were markedly reduced, while both MSCs and IL10-MSCs treatments could increase the O_2_ consumption and CO_2_ release. IL10-MSCs treatment could greatly increase O_2_ consumption and CO_2_ release compared with MSCs treatment (*p* < 0.05). There were no significant differences in RER among 4 groups (Fig. 3I). These results clearly showed that IL10-MSCs markedly alleviated the HFD-induced weight gain through promoting the energy consumption.

### IL10-MSCs treatment lowering the lipid levels of plasma and liver

**Fig. 4:**
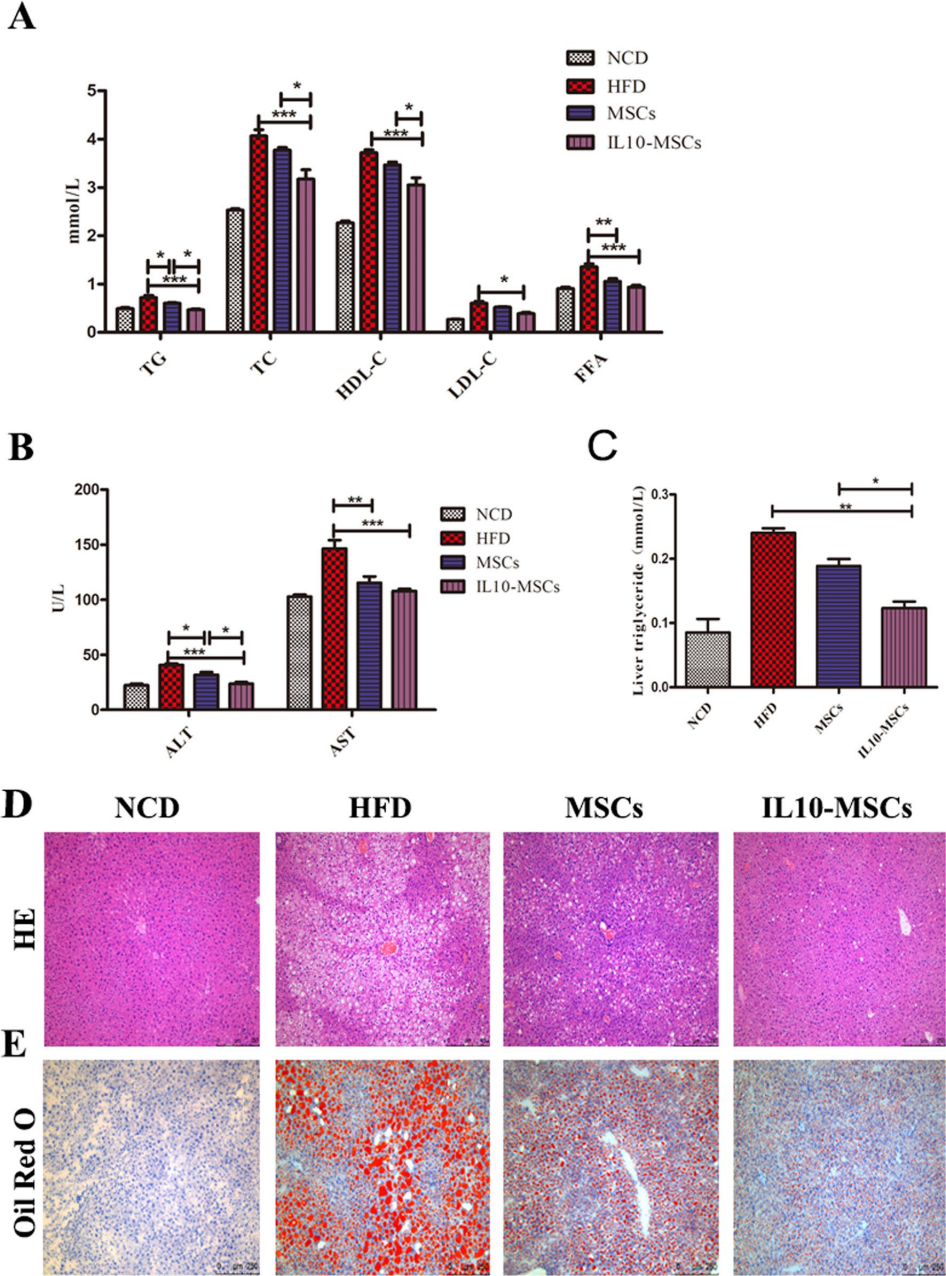
IL10-MSCs had better protective effect on lipid metabolic syndrome and liver function in HFD-fed mice (N = 6 in each group). **A** The effects of MSCs and IL10-MSCs treatment on serum lipid profiles in HFD-fed mice. **B** The serum levels of ALT and AST in four groups. MSCs and IL10-MSCs both decreased the high levels of ALT and AST induced by HFD feeding, latter had better decrease. **C** IL10-MSCs treatment clearly decreased the HFD-induced high level of hepatic TG. **D-E** H&E and Oil Red O staining of liver sections were observed. HFD feeding led to obvious lipid droplets accumulation and more white vacuoles compared with NCD. MSCs and IL10-MSCs administrations remarkably lessened vacuoles and lipid droplets in liver. Moreover, IL10-MSCs had better protective effect on decreasing vacuoles and lipid droplets accumulation. (N = 6 mice per group).* *p* < 0.05; ** *p* < 0.01; ***p < 0.001

Obesity is also the most common cause of dyslipidemia. Abnormalities in blood lipids including triglycerides (TG), total cholesterol (TC), high/low density lipoproteins (HDL-C/LDL-C), and free fatty acids (FFA) are common signs of metabolic syndrome [41, 42].We found that HFD feeding indeed induced a significant increase in the blood lipids including TG, TC, HDL-C, LDL-C, and FFA (Fig. 4A). MSCs treatment decreased the HFD-induced TG and FFA in serum, while IL10-MSCs treatment had a better decrease in serum levels of TG, TC, HDL-C, LDL-C and FFA compared with MSCs treatment. It has been well known that obesity could increase the serum lipid levels and injure the liver function. Alanine aminotransferase (ALT) and aspartate aminotransferase (AST) in serum are important indexes of liver function and can reflect the degree of liver injury [43–47]. ALT and AST are produced in hepatic cells and participate in the catalysis of protein and sugar transformation. When liver tissue is damaged, ALT and AST in hepatocytes enter the blood through the damaged membrane. We isolated the serum from mouse and assayed the levels of ALT and AST. The results showed that HFD feeding could significantly increase the levels of ALT and AST in serum, indicating that HFD-induced obesity led to liver injury. MSCs and IL10-MSCs administrations both could deeply reduce the serum levels of ALT and AST; moreover, IL10-MSCs had a stronger inhibitory effect on serum AST and ALT levels in obese mice (*p* < 0.05) (Fig. 4B). We further found that HFD could obviously increase the content of TG in the mice liver tissues. IL10-MSCs, not MSCs treatment significantly lowered the HFD-induced high level of TG in liver (*p* < 0.05) (Fig. 4C).

In HFD-induced obese patients, liver lipid production is greatly increased, and lipid decomposition is deeply reduced, which synergistically leads to accumulation of liver lipids and further causes a series of liver metabolic diseases such as nonalcoholic fatty liver (NAFLD) [48, 49]. H&E and Oil Red O staining were performed to evaluate the degree of liver fatty accumulation. The results showed that there were almost no lipid droplets in the liver of NCD animals, but obvious lipid droplets and more white vacuoles were observed in HFD group. MSCs-treated and IL10-MSCs-treated animals had remarkably lessened liver vacuoles and lipid droplet accumulation, moreover IL10-MSCs treatment had much fewer vacuoles and lipid droplet accumulation compared with MSCs treatment (Fig. 4D-E). These results revealed that IL10-MSCs treatment had a protective effect on the damage of liver function in HFD-fed mice.

### IL10-MSCs treatment significantly reducing hyperglycemia and improving insulin sensitivity in obese mice

**Fig. 5:**
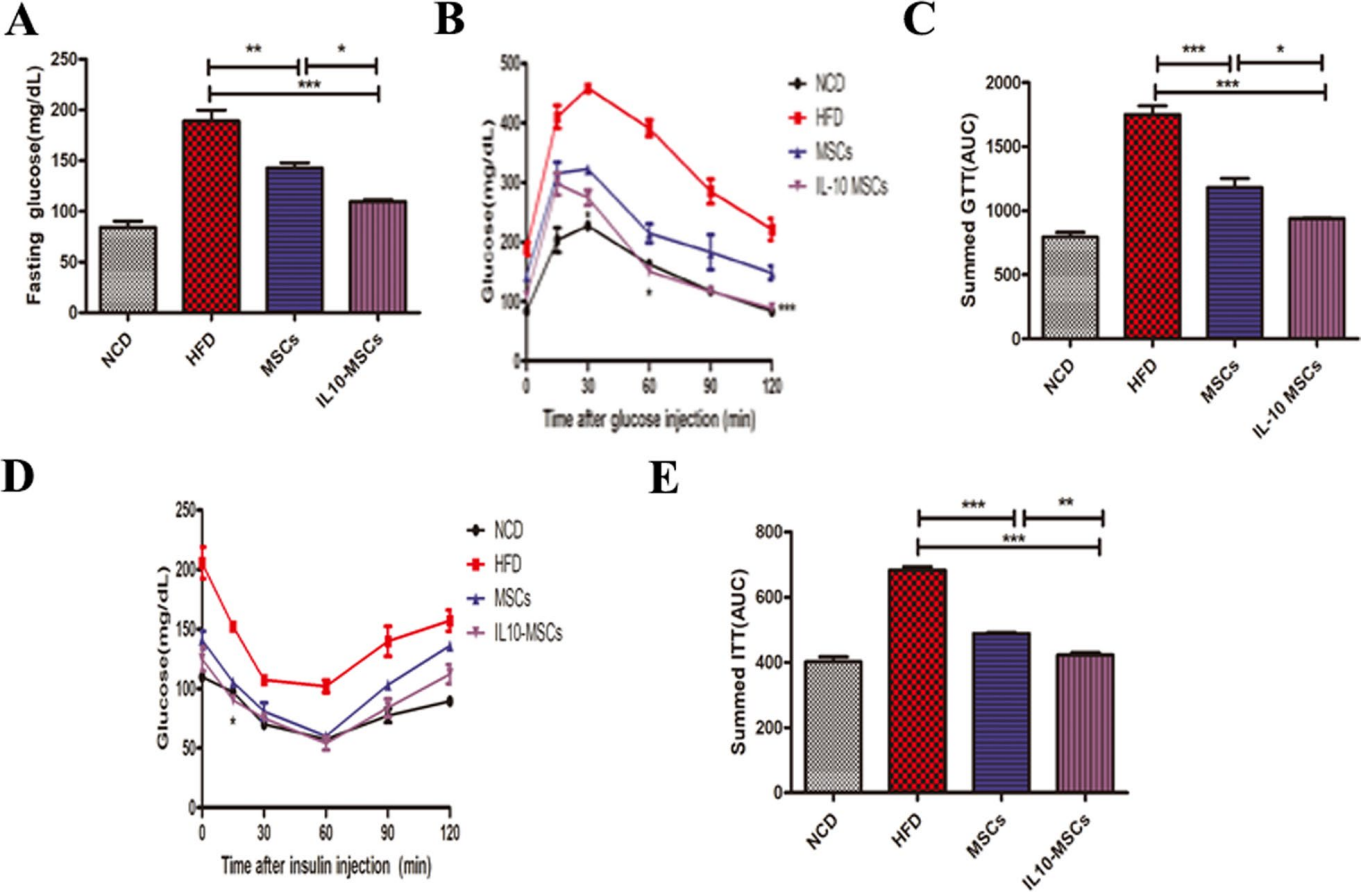
IL10-MSCs improved glucose tolerance and insulin sensitivity in HFD-fed mice. **A** HFD feeding increased the fasting blood glucose in mice, which were markedly alleviated by MSCs and IL10-MSCs treatments, especially latter. **B** The curve of blood glucose concentration at indicated time points after intraperitoneal injection of glucose. **C** Summed GTT was evaluated according to AUC of Fig. 5B. HFD feeding increased the blood glucose levels, which was reduced by MSCs and IL10-MSCs treatments. **D** The curve of blood glucose level at indicated time points after intraperitoneal injection of insulin.**E** ITT was evaluated based on AUC of Fig. 5D. (N = 6 mice per group) (* *p* < 0.05; ** *p* < 0.01; ***p < 0.001)

Obesity commonly affects the glucose homeostasis. Fasting blood glucose, GTT, and ITT are currently recognized important indicators for the diagnosis of diabetes. In order to further explore the effects of MSCs and IL10-MSCs treatments on glucose homeostasis in animals, we assayed fasting blood glucose, GTT, and ITT. After fasting for 6 h, the tail vein blood of the mice was collected to measure the fasting blood glucose value. As shown in Fig. 5A, consistent with the previous reports, the HFD feeding markedly increased fasting blood glucose of the animals compared with NCD. And MSCs and IL10-MSCs treatments both decreased the fasting blood glucose levels in HFD-fed mice, and IL10-MSCs had a greater decrease in fasting blood glucose compared with MSCs (Fig. 5A). The glucose tolerance of the mice was evaluated by intraperitoneal injection of glucose and then measuring the blood glucose levels at the corresponding time points. Compared with NCD animals, the glucose tolerance of HFD animals was unusually worse. MSCs-treated and IL10-MSCs-treated animals had remarkably increased glucose tolerance. The blood glucose of IL-10MSCs-treated animals at 60 and 120 min was lower than that of MSCs-treated animals (*p* < 0.05) (Fig. 5B). Interestingly, IL10-MSCs treated animals showed a glucose tolerance similar to that of NCD animals. The area under curve (AUC) of Fig. 5B represented the glucose tolerance in mice among 4 groups. The AUC of HFD animals increased significantly, while MSCs and IL10-MSCs treatments diminished the HFD-induced AUC (*p* < 0.01). More importantly, IL10-MSCs treatment remarkably decreased the AUC than MSCs treatment (*p* < 0.05). The above results indicated that IL10-MSCs treatment could more excellently inhibit the hyperglycemia induced by HFD in mice and extensively alleviated the impairment in glucose tolerance (Fig. 5C).

Obesity could lead to a decrease in insulin sensitivity. We next explored whether IL10-MSCs treatment could recover the damaged insulin sensitivity in HFD-fed mice. The blood glucose levels in mice were measured at indicated time points after insulin injection. From 0 to 30 min, the blood glucose in 4 groups fast decreased. Except for HFD group, the blood glucose in other 3 groups kept decreasing from 30 to 60 min after insulin injection and then the blood glucose in all groups began to increase (Fig. 5D). The AUC of Fig. 4D represented the insulin resistance. As shown in Fig. 5E, HFD-fed mice had a higher insulin resistance than NCD mice, while MSCs and IL10-MSCs treatments decreased the HFD-induced insulin resistance (*p* < 0.001). In particular, IL10-MSCs treated mice had a more decrease in insulin resistance compared with MSCs treated mice, similar to NCD mice, indicating IL10-MSCs could significantly improve insulin sensitivity in HFD mice, close to basal level.

### IL10-MSCs treatment markedly reducing the lipid accumulation and inflammatory response in adipose tissue

**Fig. 6:**
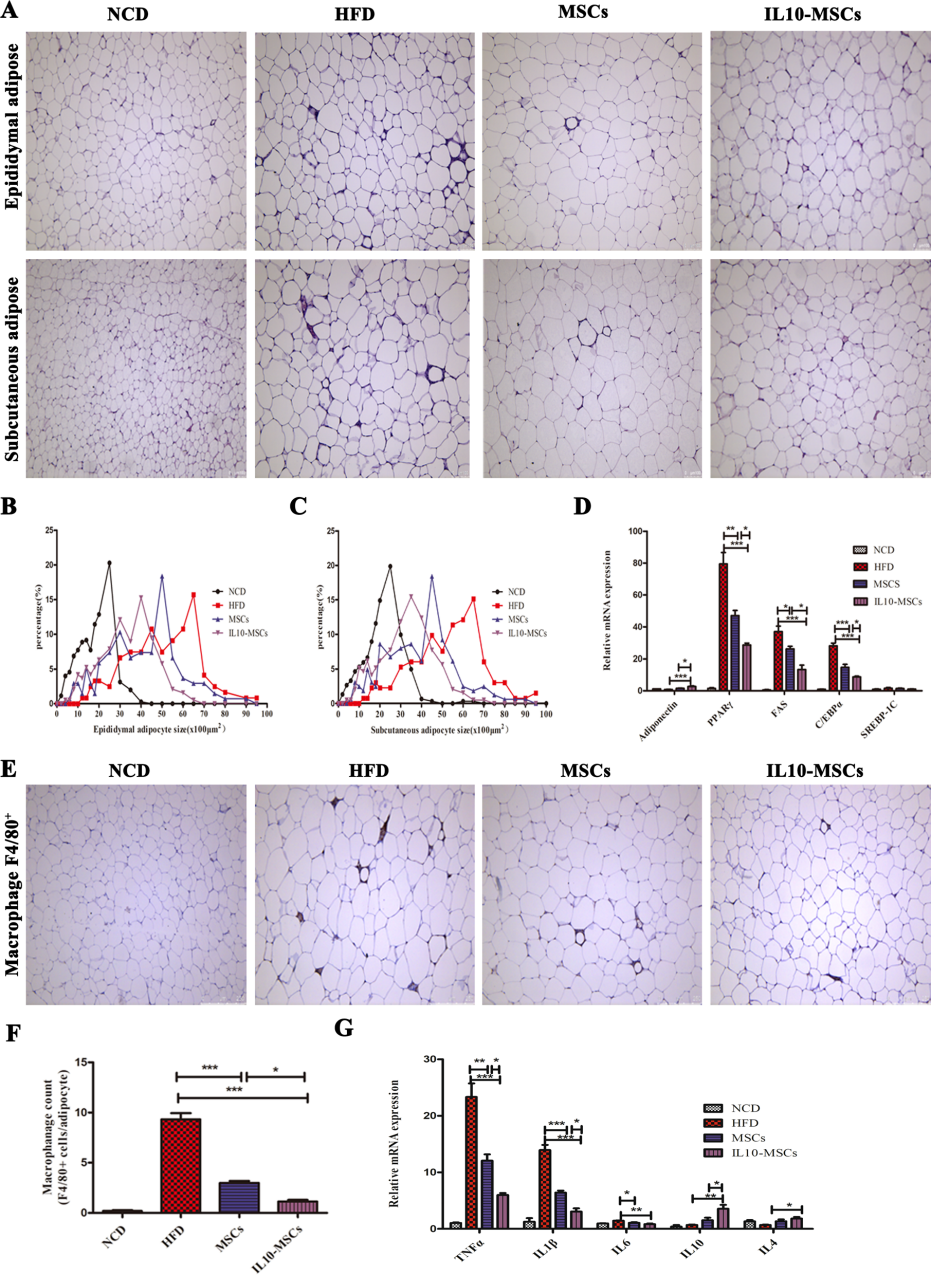
IL10-MSCs treatments repressed obesity-induced lipid accumulation and inflammation in white adipose tissue. **A** The H&E of epididymal and subcutaneous white adipose tissues. **B-C** The sizes of epididymal and subcutaneous adipocytes were analyzed by Image J software. **D** The mRNA expressions of genes regarding adipose synthesis were analyzed by RT-PCR. **E–F** The immunostaining of infiltrating macrophages (F4/80 positive cells) showed HFD feeding significantly increased the aggregations of F4/80 positive macrophages in adipose tissue, which was alleviated by MSCs and IL10-MSCs treatment, especially by latter. **G** The transcription levels of TNFα, IL1β, IL6, IL4, and IL10 in white adipose tissue were detected by RT-PCR. HFD feeding significantly promoted the transcription levels of TNFα, IL1β, and IL6, while decreased the transcription levels of IL4 and IL10. MSCs and IL10-MSCs treatments could inhibit the expressions of HFD-induced pro-inflammatory factors and increased the anti-inflammatory factors. (N = 6 per group). (* *p* < 0.05; ** *p* < 0.01; ***p < 0.001)

When body uptakes too much carbohydrates and fat and consumes less, the excess energy is converted into fat and stored, resulting in the adipocytes hypertrophy in various organs, which attributed to obesity and related chronic disorders. White adipose tissues were mainly distributed around the viscera (mainly in the epididymis) and subcutaneous tissue. To investigate whether IL10-MSCs treatments could regulate the adipocytes hypertrophy to relieve the HFD-induced obesity, we isolated mouse epididymis and subcutaneous adipose tissues and performed H&E staining. The statistical results showed that HFD feeding induced a significant increase in the size of adipocyte compared with NCD feeding (Fig. 6A-C). However, both MSCs and IL10-MSCs treatments could inhibit the hypertrophy of adipocytes; especially, IL10-MSCs treatment had a stronger inhibition in adipocyte hypertrophy. A variety of genes such as adiponectin, PPARγ, FAS, C/EBPa, and SREBP-1c takes part in synthesis of adipose tissues [50, 51]. Additionally, adiponectin, a multifaceted adipokine, controls lipid metabolism and glucose disposal in adipose tissues, exhibits antidiabetic and anti-inflammatory effects, and also has the effect of acting as an insulin sensitizer, which is secreted mainly by white adipose tissue [52].The RT-PCR method was used to detect the transcriptions of adiponectin, PPARγ, FAS, C/EBPa, and SREBP-1c in white adipose tissue. Compared with NCD animals, HFD animals had markedly higher transcription levels of PPARγ, FAS, and C/EBPa in white adipose tissues, while had a decrease in transcription level of adiponectin. At the same time, MSCs and IL10-MSCs treatments decreased the HFD-induced high transcription levels of PPARγ, FAS, and C/EBPa, as well as increased the transcription level of adiponectin in white adipose tissues. There was no significant difference in SREBP-1c transcription expression among 4 groups (Fig. 6D). These results indicated that IL10-MSCs treatment could inhibit fat synthesis and stimulate adiponectin secretion to increase fat combustion, energy consumption in adipose tissue.

There is positive correlation between obesity and infiltrated macrophages in fat tissue. In obese animals, macrophages in fat tissue often aggregate surround adipocytes, especially in extremely obese animals [53]. In order to identify and quantify ATMs, we performed immunohistochemical staining on paraffin sections against anti-F4/80 antibody, a specific marker for mature macrophages. The F4/80 positive cells in lean mice were uniformly small and scattered and rarely appeared in the form of aggregates in adipose tissues. Compared with NCD, HFD feeding significantly increased the percentage of F4/80 positive macrophages in adipose tissue, which were alleviated by MSCs and IL10-MSCs treatment, especially by latter (Fig. 6E-F). Obesity often causes chronic tissue inflammation, which in turn accelerates the further development of obesity and insulin resistance. We had observed that IL10-MSCs treatment had a protective effect on obesity and insulin resistance in HFD-fed mice, so we further explored whether IL10-MSCs treatment could alleviate chronic inflammation under HFD feeding. Inflammatory factors such as IL1β, IL6, and TNFα can stimulate inflammation, while IL4 and IL10 are classic anti-inflammatory cytokines that can suppress cellular immune response [53]. RT-PCR method was used to detect the transcription levels of TNFα, IL1β, IL6, IL4, and IL10 in white adipose tissue, for exploring the potential protective effect of IL10-MSCs treatment under HFD feeding. Compared with NCD, HFD feeding could significantly promote the transcription levels of TNFα, IL1β, and IL6, while decrease the transcription levels of IL4 and IL10, indicating the inflammatory responses were activated by HFD in white adipose tissue. MSCs and IL10-MSCs treatments had a certain inhibitory effect on expressions of HFD-induced pro-inflammatory factors and increased the anti-inflammatory factors. Especially IL10-MSCs treatment had a more obvious inhibitory effect on inflammation induced by HFD feeding (*p* < 0.05) (Fig. 6G).

### IL10-MSCs downregulating activation of MAPK JNK signal pathway induced by HFD in adipose tissues

**Fig. 7:**
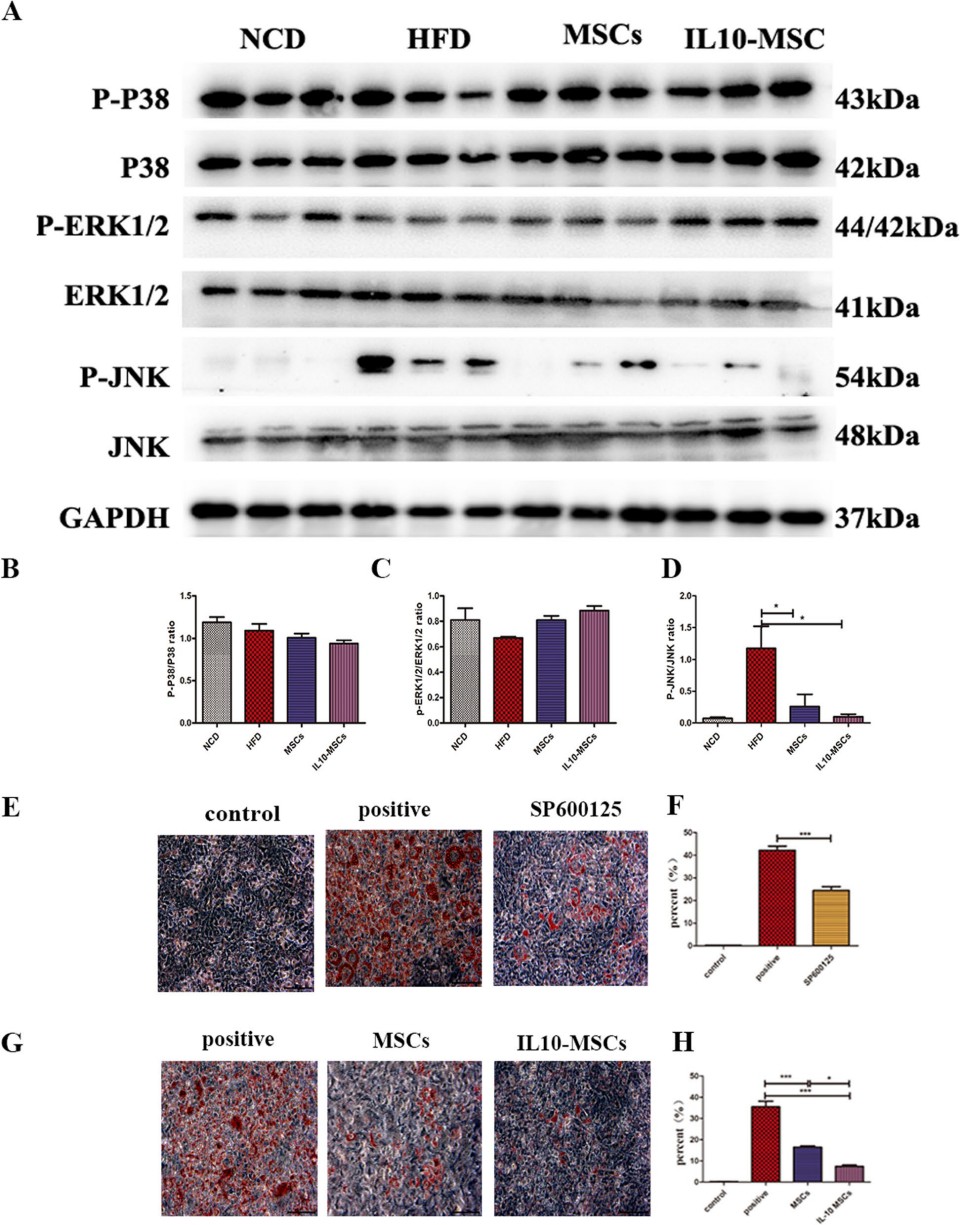
IL10-MSCs inhibiting MAPK JNK signaling in HFD-induced obese mice and reducing adipocyte differentiation in 3T3-L1 adipocytes. **A** The phosphorylations of MAPK p38, ERK1/2, and JNK in visceral white adipose tissues were detected by western blot analysis. **B-D** The quantified data of MAPK p38, ERK1/2, and JNK phosphorylation were analyzed by Image J, respectively. The phosphorylation of JNK was significantly activated in HFD-induced obese mice. MSCs, especially IL10-MSCs administration decreased the JNK phosphorylation level. **E–F** Blocking of JNK signaling by SP600125 inhibited the adipocyte differentiation in 3T3-L1 adipocytes. **G-H** Condition medium from MSCs and IL10-MSCs effectively inhibited the adipocyte differentiation in 3T3-L1 adipocytes (N = 3 per group) (*p < 0.05; **p < 0.01; ***p < 0.001).

Blocking MAPK activation can reduce the production of pro-inflammatory factors such as IL1β, IL6, etc., and decrease tissue damage and inflammation. MSCs and IL10-MSCs treatments both could inhibit the HFD-induced activation of JNK, and IL10-MSCs had a better inhibitory effect than MSCs (Fig. 7A–D). Further we investigated whether JNK signal took part in abnormal lipid accumulation in obese mice. 3T3-L1 adipocytes are common cell lines for adipocyte differentiation. JNK inhibitor (SP6001255, 2 μg/ml) was used to block JNK activation in 3T3-L1 adipocytes upon adipose differentiation medium. Results showed that JNK inhibition by SP600125 led to obvious inhibition of adipose differentiation by Oil Red O staining (Fig. 7E, F). We further explored whether MSCs and IL10-MSCs could inhibit adipocyte differentiation in 3T3-L1. As our expected, the conditioned mediums from MSCs and IL10-MSCs cultures could inhibit the differentiation of 3T3-L1 adipocytes. Importantly, the inhibitory effect of IL10-MSCs was more obvious (Fig. 7G, H). These results showed that IL10-MSCs could inhibit chronic inflammation in obese mice fed HFD by downregulating JNK signaling and reduce adipocyte differentiation and lipid droplet accumulation.

## Discussion

Obesity is becoming a more and more severe global health problem. As well known, HFD-induced murine obesity model has been extensively used in metabolic diseases [54, 55].In our study, we also found that HFD-induced obesity was indeed a consequence of systemic and progressive inflammation and demonstrated that the multiple administrations of MSCs overexpressing IL10 could be used as a potential clinical treatment against obesity and obesity related metabolic syndrome. Multiple MSCs, especially IL10-MSCs transplantations, could counteract the adverse effects of HFD on body weight, adipose tissue inflammation, lipid metabolism, and systemic insulin resistance in mice. Our results were similar to several prior studies which have addressed the metabolism in IL10-deficient mice. Clementi et al. and den Boer et al. found that HFD-fed IL10 ^–/–^ mice increased body weight and hepatic triglycerides than WT mice [56].In addition, the accumulation of adipose tissue macrophages and inflammation are the key processes in the development of systemic insulin resistance caused by obesity [57, 58]. Our study also showed that IL10-MSCs treatment decreased the level of macrophages in adipose tissue and increased insulin sensitivity. We further found that IL10-MSCs treatment downregulated the activation of MAPK JNK in adipose tissue of mice induced by HFD in vivo and thereby reduced adipose tissue inflammation. Blocking of JNK signaling by chemical inhibitor markedly inhibited the adipocyte differentiation in 3T3-L1 adipocytes in vitro and conditioned medium from IL10-MSCs also had similar activity. Therefore, our results proved that multiple IL10-MSCs transplantations could be used as an effective treatment for obesity and obesity-induced insulin resistance.

HFD-induced obesity is recognized as a low-degree chronic inflammation, which increased pro-inflammatory cytokines in multiple organs such as adipose tissue, liver, and muscle [59]. Excessive fat cell hypertrophy and increased macrophages infiltration in adipose tissue promoted various inflammatory responses in adipose tissue, abnormal secretion of adipokines and inflammatory factors, thereby exacerbating the development of obesity and obesity-related metabolic syndrome such as insulin resistance and lipid accumulation in the liver and fat [46, 47]. In obese patients, pro-inflammatory factors are overexpressed in adipose tissue, accompanied by macrophage infiltration into adipose tissue [10]. Accordingly, we supposed effectively inhibiting or reducing the occurrence of this inflammation could alleviate obesity and obesity-related metabolic diseases. MSCs have been used for the treatment of obesity because of their various advantages. The therapeutic effects of MSCs are supposed to be due to their immunomodulation and anti-inflammatory properties via soluble factors, which were extensively studied both in vitro and in vivo for the treatment of inflammatory disease [60]. In the current studies, it had been demonstrated that the intravenous administration of MSCs could ameliorate hyperglycemia and glucose intolerance in HFD-induced diabetic mice [61–63]. Previous studies have reported that MSCs could secret multifaceted cytokines, growth factors, and chemokines such as IL10, PGE2, TGF-β1,EGF, BFGF, and HGF that are helpful for anti-inflammation and anti-obesity via a paracrine mechanism [64–67].In this study, MSCs moderately downregulated the phosphorylation level of JNK and reduced the expression levels of inflammatory cytokines such as IL6, IL1β, and TNFα. Our results suggested that the positive benefits derived from MSCs alone on HFD-induced obesity might be closely associated with the inhibition of JNK signaling pathway. However, for the treatment of obesity, MSCs transplantation alone is far from the ultimate goal that could partially but not completely reverse obesity-related metabolism disorder, especially inflammation induced by HFD. With the discovery of gene therapy important for treating different disease, genetically modified MSCs can improve their therapeutic potential [68]. Rene et al. had found that both endogenous and exogenous IL10 could strongly inhibit the synthesis of TNFα, IL1β, IL6, and other factors at the transcriptional level, thereby playing an anti-inflammatory effect and leading to a reduction in obesity [69, 70]. However, due to the short half-life of recombinant IL10, it was difficult to maintain a relatively stable concentration in the body when applied to human [71]. Exerting long-term anti-inflammatory and immune effects of IL10 in vivo is a potential strategy for a variety of disorders [72, 73]. MSCs can migrate well to the surrounding inflamed tissues such as liver and adipose due to their good chemotaxis and paracrine effects and then release the anti-inflammatory factor IL10 [74, 75]

. Thus, MSCs are especially suitable as carriers for delivering desired key target proteins such as IL10 or other anti-inflammatory factors which are potentially beneficial to treat obesity and its related metabolic diseases. As expected, IL10-MSCs treatment in the current study increased the expression of anti-inflammatory markers (IL4, IL10) while significantly decreased the expression of pro-inflammatory factors(TNFα, IL1β, and IL6)and infiltration of F4/80^+^macrophages in adipose tissues. More importantly, IL10-MSCs treatment reduced the level of inflammation, which improved the insulin sensitivity of HFD-induced mice. These results suggested that IL10-MSCs had a better anti-inflammatory treatment effect on HFD-induced obesity and insulin resistance compared with naive MSCs.

Obesity is a chronic, progressive, and systemic inflammatory processes. It has been reported that in HFD-induced obesity animal models, a single transplantation of MSCs exhibits short-term effects [76].In many clinical studies, patients were treated with multiple MSCs transplantations to obtain positive results [77, 78].Multiple injections could provide more living cells that remodel the injured site environment to promote regeneration [79].Therefore, multiple intravenous IL10-MSCs transplantations may be necessary. In this study, after CM-Dil-labeled IL10-MSCs injected, red fluorescent signals could be detected from day 3, 6, and 14 in vivo. To further test the survival of transplanted CM-Dil-labeled IL10-MSCs in vivo, TUNEL staining with green FITC was performed. The results showed that the red fluorescence of most CM-Dil-labeled IL10-MSCs in liver and lung did not coincide with the green fluorescence of apoptotic cells indicating there were few apoptotic IL10-MSCs. Therefore, the present finding had been proved that IL10-MSCs could survive in vivo for at least 14 days, but a significant decrease in fluorescence was detected at day 14. Therefore, based on the results of grafted MSCs tracing, we performed IL10-MSCs therapy through intravenous administration every 14 days, which could continuously release endogenous IL10 in vivo and played a sustained anti-inflammatory effect to treat HFD -induced obesity, leading to better therapeutic effects.

In our study, after 12-week HFD feeding, mice demonstrated substantial increase in body weight. This was associated with significantly elevated fat and liver weight. In contrast, IL10-MSCs treatment obviously decreased body weight. Thus, it was essential to investigate whether IL10-MSCs treatment decrease energy intake or increase energy expenditure. Therefore, we measured energy metabolism in mice through metabolic cage examining. We observed that food intake was not significantly changed in HFD-induced mice. But the expenditure of VO_2_ and VCO_2_ of HFD animals was markedly reduced, while both MSCs and IL10-MSCs treatments could increase the VO_2_ and VCO_2_ to promote energy expenditure. In addition, there was similar report that hAMSCs-CM promoted energy expenditure by increasing VO_2_, VCO_2_ to reduce weight gain [80]. Additionally, most studies supported a protective role of IL10 in the regulation of metabolic inflammation and insulin resistance [81, 82]. Furthermore, IL10 expression increased in adipose tissue following weight loss and reduced proinflammatory gene expressions in obese subjects [12]. Consistent with our results, it also showed that IL10-MSCs treatment not only reduced body weight but also decreased the level of inflammation in HFD-induced obese mice.

Obesity may activate the JNK signaling pathway to regulate the expression of metabolism and inflammation-related genes and proteins [83]. JNK may activate the phosphorylation of many substrates located downstream of the JNK signaling pathway, including c-Jun, activator protein-1 (AP-1), P53, insulin receptor substrate-1 (IRS-1), etc. [84]. Activated C-Jun transcription factors bind to AP-1 sites, thereby promoting the expression of pro-inflammatory factors (e.g., TNFα, IL1β, IL6, etc.), which play a key role in impaired glucose tolerance and insulin resistance by HFD-induced obesity [85]. Gürol Tuncman et al. had reported that JNK1^−/−^mice were protected from HFD-induced obesity, inflammation, and insulin resistance, which implicated JNK was involved in obesity-related inflammation [86]. In this study, a higher-than-normal level of JNK activation was observed in HFD-induced mice and there were significantly increased expressions of pro-inflammatory factors such as IL1β, TNFα, and IL6. Importantly, IL10-MSCs treatment downregulated the phosphorylation level of JNK and had a more significant reduction in the level of inflammation in adipose tissue of HFD-feeding mice. In addition, studies have been shown that 3T3-L1 adipocytes undergo adipogenesis where JNK was upregulated during differentiation [87, 88]. Furthermore, SP600125, a selective inhibitor of the JNK pathway, could significantly reduce lipid droplet formation in 3T3-L1 adipocytes under the conditions of inducing differentiation medium. Besides IL10-MSCs decreased the activation of the JNK pathway, we also found that the supernatant of IL10-MSCs markedly reduced adipocyte differentiation and lipid accumulation in 3T3-L1 adipocytes. Therefore, our experimental results proved that IL10-MSCs might alleviate the JNK signaling pathway to reduce adipose tissue inflammation and decrease lipid accumulation in adipocytes.

## Conclusions

Our study showed that multiple intravenous transplantations of MSCs and IL10-MSCs played a positive and important role in the development of obesity diseases in alleviating obesity-associated metabolic syndromes such as reducing inflammation and lipid accumulation in adipose tissue, and combating adipocyte differentiation and increasing insulin sensitivity. Long-term inhibition of inflammation by genetically modified MSCs treatment is an effective strategy in preventing diet-induced obesity and obesity-related metabolic syndrome.

## Data Availability

In the study, all data used and/or analyzed are available from the corresponding author on reasonable request.

## Abbreviations

HFD: High-fat diet
IL10: Interleukin 10
IL6: Interleukin 6
IL4: Interleukin 4
IL1β: Interleukin1 beta
IL2: Interleukin 2
HUCMSCs: Human umbilical cord-derived mesenchymal stromal cells
TG: Triglyceride
TC: Total cholesterol
HDL-C and LDL-C: High- and low-density lipoprotein cholesterol
ALT: Alanine aminotransferase
AST: Aspartate aminotransferase
ATMS: Adipose tissue macrophages
TNFα: Tumor necrosis factor-α
AAV: Adeno-associated virus
MSCs: Mesenchymal stromal cells
MAPK: Mitogen activated protein kinases
IRS-1: Insulin receptor substrate-1
RER: Respiratory exchange ratio
GTT: Glucose tolerance test
ITT: Insulin tolerance test
H & E: Hematoxylin and eosin
RT-PCR: Reverse transcription-polymerase chain reaction
PGE2: Prostaglandin E2
TGF-β1: Transforming growth factor beta 1
EGF: Epidermal growth factor
BFGF: Basic fibroblast growth factor
HGF: Hepatocyte growth factor
Ang-1: Angiopoietin-1
